# Artificial intelligence for the diagnosis of erythematous-squamous dermatological diseases: technological contributions to primary care

**DOI:** 10.1016/j.abd.2025.501169

**Published:** 2025-08-13

**Authors:** Raiza Brito Cipriano, Wilson Falco Neto, Fabiano N. Barcellos Filho, Alexandre Dias Porto Chiavegatto Filho

**Affiliations:** a Department of Medicine, Escola Superior de Ciências, Santa Casa de Misericórdia de Vitória, Vitória, ES, Brazil; b Department of Internal Medicine, Faculdade de Medicina de Catanduva, Catanduva, SP, Brazil; c Department of Epidemiology, Faculdade de Saúde Pública, Universidade de São Paulo, São Paulo, SP, Brazil

**Keywords:** Artificial intelligence, Dermatology, Diagnosis, differential, Expert systems, Machine learning

## Abstract

**Background:**

Accurate diagnoses in dermatology can be challenging for general practitioners. In this context, the support of artificial intelligence tools can be beneficial in the Brazilian primary care setting.

**Objectives:**

To develop an interpretable machine-learning algorithm capable of assisting in the diagnosis of erythematous-squamous dermatological diseases through clinical data, without histopathological support.

**Methods:**

The random-forest algorithm was trained with the public Dermatology database of 366 patients diagnosed with: chronic dermatitis, lichen planus, pityriasis rosea, pityriasis rubra pilaris, psoriasis, or seborrheic dermatitis. The model was evaluated by performance metrics and interpretability techniques.

**Results:**

The model showed good predictive performance, with ROC-AUC ranging from 0.89 to 1.00, and overall accuracy of 0.86. The best results were for the diagnosis of pityriasis rubra pilaris (f1-score: 1.00) and the worst for chronic and seborrheic dermatitis (f1-score: 0.77 and 0.76, respectively). The clinical characteristics that most influenced the model's decision were, in decreasing order: involvement of knees and elbows, involvement of scalp, Koebner phenomenon, polygonal papules, and involvement of oral mucosa.

**Study limitations:**

The model was not validated with Brazilian data.

**Conclusion:**

The developed technology obtained good predictive performance and clinical coherence. There is a need for adaptation for implementation, using national data. The results indicate the potential for similar models to be improved and adapted to clinical practice for the benefit of the Unified Heath System.

## Introduction

The different possible diagnoses in dermatology are often complex and require physicians to go through a long learning curve to make assertive diagnoses. A study comparing the performance of general practitioners and dermatologists in general clinical dermatology cases demonstrated agreement of only 45% of diagnoses in cases without support from histopathological data.^1^ Another study, a systematic review with meta-analysis on medical efficacy in the diagnosis of melanoma, demonstrated a sensitivity of 81% for dermatologists, while primary care general practitioners had a sensitivity of only 42%.^2^ In a similar vein, erythematous-squamous dermatological diseases, such as dermatitis, psoriasis, and lichen planus, can be difficult to differentiate and diagnose because they have very similar characteristics, especially for non-specialist physicians.

Machine learning models can be a useful tool to aid in diagnosis for physicians, especially general practitioners in primary care in the Brazilian Unified Health System (SUS, *Sistema Único de Saúde*), where there would be a greater concentration of dermatological diagnostic errors due to their generalist nature, with quick consultations and, in many cases, without the support of complementary exams. Some models have already been developed around the world for the diagnosis of erythematous-squamous dermatological diseases that have shown excellent performance, using clinical and histopathological data to predict diagnoses.^3^ However, in scenarios where histopathological diagnosis is not possible or is time-consuming, tools that improve diagnostic accuracy without the support of complementary exams can optimize clinical practice, enabling more assertive treatments.

This study aimed to develop an interpretable machine-learning algorithm capable of differentiating and diagnosing six erythematous-squamous dermatological diseases, based on clinical data, without histopathological support.

## Method

### Database and preprocessing

The “Dermatology” database of erythematous-squamous dermatological diseases was used, which contains individualized data without patient identification. The data were originally made available through a partnership between the Department of Dermatology, Gazi University School of Medicine, and the Department of Computer Engineering and Computer Science, Bilkent University, and are freely available on the UC Irvine Machine Learning Data Repository.^4^ Therefore, the present study does not require approval by a Research Ethics Committee.

The database contains clinical and histopathological data from 366 patients diagnosed with one of the six diseases under study: chronic dermatitis (n = 52), lichen planus (n = 72), pityriasis rosea (n = 49), pityriasis rubra pilaris (n = 20), psoriasis (n = 112), and seborrheic dermatitis (n = 61). The variables with clinical attributes are: erythema, desquamation, defined borders, pruritus, Koebner phenomenon, polygonal papules, follicular papules, involvement of the oral mucosa, involvement of knees and elbows, family history and age. The histopathological variables are: pigment incontinence, eosinophilic infiltrate, neutrophil infiltrate, papillary dermal fibrosis, exocytosis, acanthosis, hyperkeratosis, parakeratosis, widening of the epidermal ridges, elongation of the epidermal ridges, thinning of the suprapapillary epidermis, spongiform pustule, Munro's microabscess, focal hypergranulosis, the disappearance of the granular layer, vacuolization and damage to the basal layer, spongiosis, epidermal ridges in a “sawtooth pattern”, follicular horny plug, perifollicular parakeratosis, mononuclear inflammatory infiltrate and band infiltrate.

Since the histopathological analysis, combined with clinical history, can be considered the “gold standard” in the diagnosis of erythematous-squamous dermatological conditions, it was decided to train the model without the support of these variables for prediction. The choice was made with the aim of developing a model that could assist clinical practice, which sometimes does not have access to histopathology.

Data preprocessing consisted of eliminating columns of histopathological data and patients with incomplete clinical data. The final set consisted of 359 patients, 98% of the original 366 evaluated patients.

### Model and performance metrics

A machine learning model for Multiclass Classification was developed using the Random Forest algorithm and the “One-vs.-Rest” strategy, in Python via the Google Colab™ platform. The Holdout Method was used, in which the model was trained by dividing the data between training and testing subsets, in which 70% of these were used for learning the algorithm and 30% for testing and evaluating the predictive performance of the model on new data.

In the Random Forest model used, 100 trees were generated, with an average depth of 13.21; ranging from a maximum depth of 17 to a minimum of 11. For each node, a maximum number of features was selected based on the square root of the total number of available features (“sqrt”). The impurity criterion used was the Gini index, and the minimum number of samples per leaf was defined as 1, while the minimum number of samples required to divide a node was 2.

To assess the predictive performance, a Confusion Matrix was generated and the area under the Receiver Operating Characteristic Curve (ROC-AUC), sensitivity, specificity, Positive Predictive Value (PPV), Negative Predictive Value (NPV), F1-score and model accuracy were calculated.

### Interpretability

The model interpretation technique chosen was the SHAP (SHapley Additive exPlanations) method, which is based on the Shapley Values ​​technique.^5^ In this case, the objective is to explain the predictions of the machine learning model based on the contribution of each variable to the prediction of the final result.^6^ In this study, the variables are the clinical characteristics of each patient and the final result, or prediction, is the diagnosis of one of the erythematous-squamous diseases in the study.

Thus, each clinical characteristic receives a SHAP value, indicating the impact of this variable on the diagnosis prediction. If the SHAP value of a variable is zero, it means that the presence or absence of that clinical characteristic does not influence the diagnostic decision made by the model. If the SHAP value is positive, the presence of the characteristic has an influence in favor of the diagnosis. On the other hand, if the SHAP value is negative, it is interpreted that the presence of the characteristic has an influence contrary to the diagnosis. Finally, high SHAP values ​​(in modulus) indicate that the influence of the variable on the prediction is high, while low SHAP values ​​(in modulus) indicate that the variable has little influence on the disease prediction.^6^

## Results

### Algorithm performance

**Figure 1:**
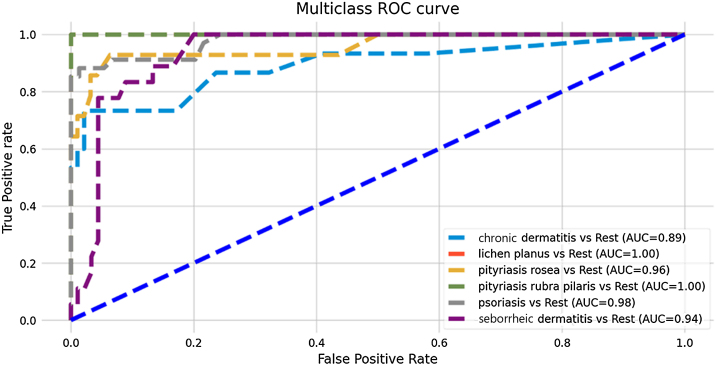
ROC curves of the Random Forest model for multi-class classification and values ​​of their respective Areas Under the Curve (AUC).

The developed model showed good predictive performance, even without the support of histopathological variables (overall accuracy of the model: 86%), as shown in Table 1. The most assertive diagnoses were made in the predictions of the diseases pityriasis rubra pilaris and pityriasis rosea (sensitivity: 100% and 93%, respectively), while chronic dermatitis and seborrheic dermatitis attained the lowest metrics (sensitivity: 67% and 78%, respectively). Nevertheless, the model was successful in predicting negative results for these diseases, achieving high specificity and NPV values ​​(chronic dermatitis: 99% and 94%; seborrheic dermatitis: 94% and 96%). Fig. 1 shows the ROC curves resulting from the predictive analysis of the test data.

**Table 1:** Individual and overall measures of diagnostic model performance.

Disease	ROC-AUC	Sensitivity	Specificity	PPV	NVP	F1-score	Overall Model Accuracy
Chronic dermatitis	0.89	0.67	0.99	0.91	0.94	0.77	0.86
Lichen planus	1.00	0.90	1.00	1.00	0.98	0.95	
Pityriasis rosea	0.96	0.93	0.94	0.68	0.99	0.79	
Pityriasis rubra pilaris	1.00	1.00	1.00	1.00	1.00	1.00	
Psoriasis	0.98	0.91	0.96	0.91	0.96	0.91	
Seborrheic dermatitis	0.94	0.78	0.94	0.78	0.96	0.76	

ROC-AUC, Receiver Operating Characteristic - Area Under Curve; PPV, Positive Predictive Value; NVP, Negative Predictive Value.

**Figure 2:**
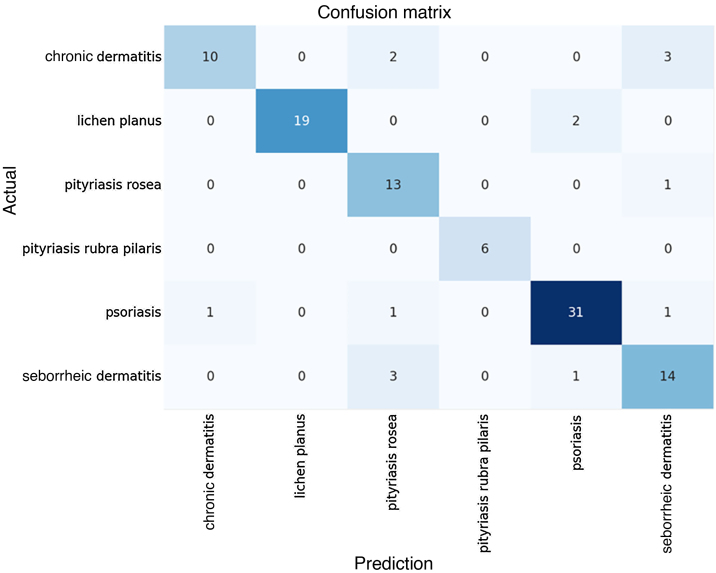
Confusion matrix.

The algorithmic performance results become clear when analyzed together with the Confusion Matrix of predicted versus actual results, shown in Fig. 2 below.

### Model interpretability: the importance of clinical variables

For a general analysis, the characteristics were classified in decreasing order of influence on the model's decisions, using the average absolute impact values ​​(average SHAP).

**Figure 3:**
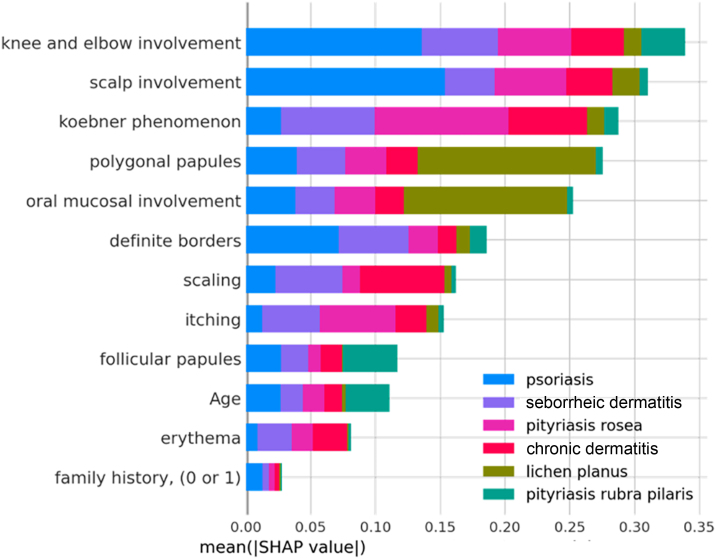
SHAP Summary Plot: overall importance of variables for predicting outcomes and contribution to specific predictions.

The clinical characteristics that most influenced the differentiation between the analyzed skin diseases – that is, those with the highest average SHAP – were: involvement of knees and elbows, involvement of the scalp, Koebner phenomenon, polygonal papules, involvement of the oral mucosa and defined borders, as observed in Fig. 3 below. The characteristics “family history”, “erythema” and “age” had little influence on the general diagnosis.

It is also possible to observe how much each characteristic contributes, in absolute terms, to each diagnosis; for example: the variables “involvement of knees and elbows” and “involvement of scalp” have a very significant influence on the diagnosis of psoriasis (blue bars), while the variables “polygonal papules” and “involvement of oral mucosa” had a greater influence on the diagnosis of lichen planus (green bars). Thus, the diseases associated with the best predictive performances of the model are those that have unique distributions of mean SHAP values. Likewise, diseases with similar distributions of mean SHAP values ​​among the variables, such as when comparing chronic and seborrheic dermatitis (red and purple bars, respectively), have the worst predictive performances, since the model may have difficulty differentiating them.

**Figure 4:**
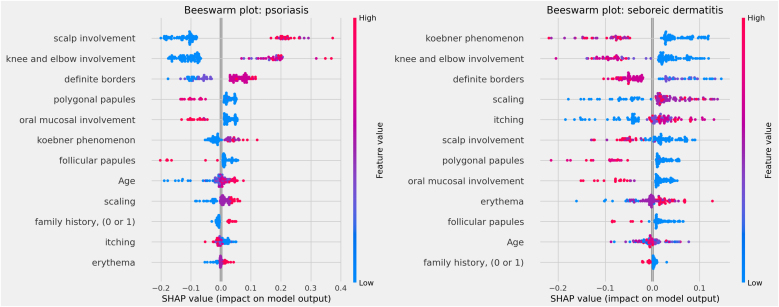
Beeswarm Plots: psoriasis (left) and seborrheic dermatitis (right).

**Figure 5:**
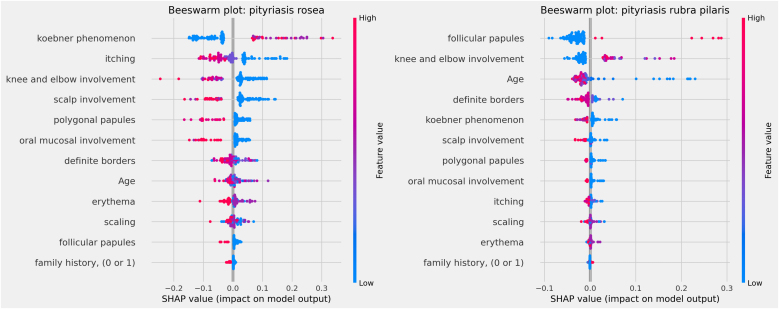
Beeswarm Plots: pityriasis rosea (left) and pityriasis rubra pilaris (right).

**Figure 6:**
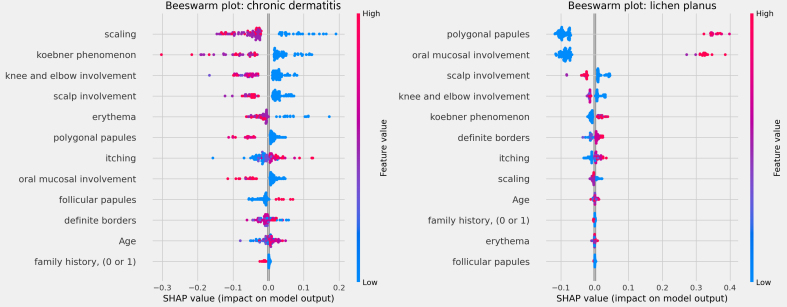
Beeswarm Plots: Chronic dermatitis (left) and lichen planus (right).

The influences of clinical characteristics in the definition of each differential diagnosis can also be observed in more detail and individually in the Beeswarm-type graphs, in Figure 4, Figure 5, Figure 6 below. In this case, it is possible to identify how the variables influenced the model decision-making process, in which the values ​​of the variables (pink tones are higher values, indicating a greater presence of the characteristic, and blue tones are lower values, indicating a lesser presence of the characteristic) correlate with the impact on the diagnostic result (positive SHAP-values ​​are favorable to the diagnosis in question, while negative SHAP-values ​​are unfavorable to the diagnosis).

Based on the interpretation of the proposed model and the clinical characteristics made available for this analysis, it can be verified that chronic dermatitis (ROC-AUC = 89%) was positively correlated mainly with pruritus and lower desquamation values, while the presence of the Koebner phenomenon had an unfavorable effect on the diagnosis. Lichen planus (ROC-AUC = 100%) was positively and significantly correlated with the presence of polygonal papules and involvement of the oral mucosa. Pityriasis rosea (ROC-AUC = 96%) correlated positively and significantly with the Koebner phenomenon, while the presence of higher values ​​of pruritus, and involvement of knees, elbows, scalp or polygonal papules had an unfavorable effect on the diagnosis. Pityriasis rubra pilaris (ROC-AUC = 100%) correlated positively and significantly with the presence of follicular papules, mean values ​​of involvement of knees and elbows, and during young age (first two decades of life). The prediction of psoriasis (ROC-AUC = 98%) was positively and significantly influenced mainly by the involvement of knees, elbows and/or scalp. Finally, seborrheic dermatitis (ROC-AUC = 94%) had its prediction positively influenced by desquamation, pruritus and erythema. On the other hand, the presence of the Koebner phenomenon, involvement of knees and elbows, and defined borders had an unfavorable effect on its diagnosis.

## Discussion

Overall, the model showed good predictive performance, showing an overall accuracy of 86%. Studies published around the world were found, predominantly in electronic engineering and technology journals, which used the same database to develop different predictive algorithms for erythematous-squamous diseases, all of which used clinical and histopathological variables to predict the diagnosis. One of these studies used Classification and Regression Tree (CART), obtained an overall accuracy of 93.69% and similar results to the present study, showing a specificity of 100% for “lichen planus” and “pityriasis rubra pilaris”, and showed better sensitivity for “seborrheic dermatitis” (100%) when compared to this study (78%).^7^ Another study also used Random Forest and showed cross-validation values ​​of 51.13%. Higher values ​​were obtained by other algorithms, such as 96.65% with Deep Neural Networks and 95.80% with XGBoost.^8^

The results found by the study demonstrate the potential for applying machine learning algorithms in dermatological diagnosis. When compared to the results of this study, without histopathological data, it is clear that the algorithm did not suffer a significant loss of performance, making it viable for clinical practice. The importance of tools like this lies in the possibility of optimizing clinical practice aiming to provide assertive treatments without the support of complementary exams.

When the interpretability of the results is verified, that is, how the variables contributed to the prediction of each diagnosis (Fig. 2), an important convergence with clinical practice can be observed. When observing the characteristics of pityriasis rubra pilaris in the medical literature, one of the main characteristics common to the disease subtypes is the presence of hyperkeratotic follicular papules. Furthermore, pityriasis rubra pilaris is diagnosed according to its subtypes, which are based on age of onset, lesion distribution and prognosis^9^ ‒ these characteristics are in line with the main findings of this study. In psoriasis, the involvement of knees and elbows is also a characteristic of the clinical diagnosis and the Koebner phenomenon demonstrated relevance, as expected.^10^ Thus, the exercise of comparing the results of this study with the medical literature can be performed for each disease analyzed.

A limitation of the model lies in its dependence on the quality of the database. In this study, the data collected from a non-Brazilian population may not be representative of the characteristics of the local population. These limitations were also verified and mitigated in the study by Wichmann et al., who developed models for predicting death from COVID-19 with data from 18 Brazilian hospitals and compared optimization strategies, finding that the best performance occurred when the model was trained locally, that is when a model was trained with data from a hospital to predict the data from the same hospital.^11^ Moreover, the quality of the data for training depends on the data collection operator, while the quality of the results depends on the end user during the clinical use of the proposed model, since an incorrectly identified clinical sign could lead to an incorrect diagnosis. Some of the characteristics of lesions used to train the model under study may require greater knowledge from the operator and user, such as the definition of the Koebner phenomenon and differentiation between papules.

Therefore, the developed technology requires adaptations for its local implementation, since the predictive capacity with data from other hospitals, especially from other continents, may present variations that impact its effectiveness. In addition to training on a local database, the model would need to be incorporated into software with an intuitive and instructive interface, to support general practitioners in characterizing the lesions according to the recommended scales. Also, the inclusion of a functionality for reading and interpreting photos of skin lesions would make the technology even more useful in non-specialized clinical practice.

## Final considerations

This study identified technological possibilities applicable to the Unified Heath System, proposing the construction of a machine learning algorithm for multiclass classification of erythematous-squamous dermatological diseases with the potential to assist in the reality of clinical practice in Brazilian public primary care. For future studies, it will be important to develop this technology to include the construction of an updated and multicenter Brazilian database, and the improvement of the prediction technique using models capable of interpreting photos of skin lesions, incorporated into software with an interface suitable for use by non-specialist physicians.

## Research data availability

The entire dataset supporting the results of this study was published in this article.

## Scientific Associate Editor

Luciana P. Fernandes Abbade.

## Financial support

None declared.

## Authors' contributions

Raiza Brito Cipriano: Design and planning of the study; analysis and interpretation of data; implementation of computer code and supporting algorithms; drafting and editing of the manuscript or critical review of important intellectual content.

Wilson Falco Neto: Drafting and editing of the manuscript or critical review of important intellectual content.

Fabiano N. Barcellos Filho: Implementation of computer code and supporting algorithms; drafting and editing of the manuscript or critical review of important intellectual content; effective participation in research orientation.

Alexandre Dias Porto Chiavegatto Filho: Drafting and editing of the manuscript or critical review of important intellectual content; effective participation in research orientation; critical review of the literature; approval of the final version of the manuscript.

## Conflicts of interest

None declared.
